# Optimization of Biodiesel Production Process Using MoO_3_ Catalysts and Residual Oil: A Comprehensive Experimental 2^3^ Study

**DOI:** 10.3390/molecules29102404

**Published:** 2024-05-20

**Authors:** Adriano Lima da Silva, Helder de Lucena Pereira, Herbet Bezerra Sales, Juliana Kelly Dionízio, Mary Cristina Ferreira Alves, Danyelle Garcia Guedes, Carlos Bruno Barreto Luna, Ana Cristina Figueiredo de Melo Costa

**Affiliations:** 1 Synthesis Laboratory of Ceramic Materials (LabSMaC), Graduate Program in Materials Science and Engineering (PPGCEMat), Federal University of Campina Grande (UFCG), Campina Grande 58429-900, Brazil; hld.lucena@gmail.com (H.d.L.P.); herbet_bezerra@hotmail.com (H.B.S.); julianakelly71@gmail.com (J.K.D.); danyelle.garcia@estudante.ufcg.edu.br (D.G.G.); ana.figueiredo@professor.ufcg.edu.br (A.C.F.d.M.C.); 2 Graduate Program in Chemistry (PPGQ), State University of Paraíba (UEPB), Campina Grande 58429-900, Brazil; mary.alves@servidor.uepb.edu.br; 3 Materials Engineering Academic Unit, Polymer Processing Laboratory, Federal University of Campina Grande, Av. Aprígio Veloso, 882, Campina Grande 58429-900, Brazil; brunobarretodemaufcg@hotmail.com

**Keywords:** planning, factorial, transesterification, esterification, industry

## Abstract

The study aimed to utilize MoO_3_ catalysts, produced on a pilot scale via combustion reaction, to produce biodiesel from residual oil. Optimization of the process was conducted using a 2^3^ experimental design. Structural characterization of the catalysts was performed through X-ray diffraction, fluorescence, Raman spectroscopy, and particle size distribution analyses. At the same time, thermal properties were examined via thermogravimetry and differential thermal analysis. Catalytic performance was assessed following process optimization. α-MoO_3_ exhibited a monophasic structure with orthorhombic phase, whereas α/h-MoO_3_ showed a biphasic structure. α-MoO_3_ had a larger crystallite size and higher crystallinity, with thermal stability observed up to certain temperatures. X-ray fluorescence confirmed molybdenum oxide predominance in the catalysts, with traces of iron oxide. Particle size distribution analyses revealed polymodal distributions attributed to structural differences. Both catalysts demonstrated activity under all conditions tested, with ester conversions ranging from 93% to 99%. The single-phase catalyst had a long life cycle and was reusable for six biodiesel production cycles. The experimental design proved to be predictive and significant, with the type of catalyst being the most influential variable. Optimal conditions included α-MoO_3_ catalyst, oil/alcohol ratio of 1/15, and a reaction time of 60 min, resulting in high biodiesel conversion rates and showcasing the viability of MoO_3_ catalysts in residual oil biodiesel production.

## 1. Introduction

Researchers worldwide have directed their efforts to explore alternative energy sources, aiming to mitigate the negative impacts of climate change. Over the past two decades, there has been a significant transition from fossil fuels to adopting more efficient and low-carbon resources in producing sustainable fuels [1,2]. Biodiesel, known as alkyl esters, is obtained through the esterification of fatty acids and the transesterification of triglycerides, including animal or vegetable fat residues. In these processes, the reaction between a lipid and an alcohol, such as ethanol or methanol, occurs in the presence of a catalyst [3].

Biodiesel has several advantages such as its biodegradability, reduced greenhouse gas emissions profile, and lower sulfur and particulate emissions [4]. As for the raw material for producing this biofuel, waste oil not only makes the process economically viable but also avoids competition with edible oils, providing an environmentally appropriate solution for the disposal of frying oil waste.

A wide range of catalysts are used in biodiesel synthesis [5], covering types such as acid [6], basic [7], homogeneous [8], heterogeneous [9], ionic [10], among others. Solid acid catalysts have advantages such as high conversion of fatty oils into biodiesel, water tolerance, reduced waste treatment costs, and production of purer glycerol. Additionally, they are easily separated, reused, and regenerated as needed, resulting in less leaching from activity sites [11,12]. Catalysts that have molybdenum [13] as the main component have aroused great interest due to their acidic properties, Lewis and Brønsted, and their ability to exist in different oxidation states. These demonstrate promise for applications in industrial catalytic processes [14].

The combustion reaction is considered a relatively simple, effective, and low-cost technique, widely established in the literature as a procedure used for the synthesis of various types of oxides [15,16,17] with applications in the production of biodiesel [18]. The innovative aspect of this work is in the consolidation of MoO_3_ catalysts obtained through the combustion reaction, offering a significant contribution to studies on the optimization of the biofuel production process.

In addition to the catalyst, factors such as temperature, time, alcohol/oil ratio, amount of catalyst, and type of oil are crucial to improving the effectiveness of esterification and transesterification reactions. Each of these elements presents opportunities for optimization, both in terms of energy and chemical consumption and in relation to the process’s environmental impact [19,20]. It is crucial to carefully plan experiments and methods to achieve maximum biodiesel yields with efficient resource utilization. Optimization techniques, including linear and non-linear equations, are highly recommended for this purpose. These techniques are advantageous because they are easy to analyze and capable of resolving complex conditions effectively [21].

Recent studies have focused on optimizing the reaction parameters of biodiesel production by heterogeneous catalysis using different methodologies [22,23] such as supervised regression; Huber regression; LASSO; RVS and RNA models [24]; response surface methodology (RSM) [25,26,27,28] metaheuristic algorithms like GA, PSO, and FA [29]; multiparametric optimization [30]; and factorial experimental design [22,24,26,29,31].

Arrais Gonçalves, Karine Lourenço Mares, Roberto Zamian, Narciso da Rocha Filho, and Rafael Vieira da Conceição [32] studied the ideal conditions for biodiesel production using the acidic heterogeneous magnetic catalyst MoO_3_/SrFe_2_O_4_. The optimal reaction conditions were established as a temperature of 164 °C, alcohol/oil molar ratio of 40:1, catalyst dosage of 10%, and reaction time of 4 h, resulting in an ester conversion of 95.4%. The catalyst showed catalytic and magnetic activity even after eight reaction cycles, suggesting its viability for future applications. de Brito, Gonçalves, dos Santos, da Rocha Filho, and da Conceição [33] used the catalyst of 30-MoO_3_/Nb_2_O_5_ in the synthesis of biodiesel by transesterification. Under optimized conditions (145 °C, 2.5 h, 20:1 methanol/oil molar ratio, and 6% catalyst), a 94.2% conversion to esters was achieved.

Zhang and Xie (2023) [13] investigated the performance of a ZrMo catalyst in FFA esterification of simulated acidic oils containing 20 wt% oleic acid. FFA conversion reached 89.3% after 1 h of reaction and increased to 94.7% when the reaction lasted 5 h. Wang et al. (2022) [20] compared transesterification activity with esterification using the MoO_3_/ZrO_2_/KIT-6 catalyst. The results showed that FFA conversion was significantly higher than oil conversion, suggesting that the catalyst catalyzes FFA esterification more efficiently than oil transesterification. The esterification conversion reached 88.5% in 1 h and increased to 96.7% in 6 h.

Based on the studies referenced and seeking to offer new perspectives on heterogeneous catalysts and more sustainable routes for the energy sector, as well as consolidating synthesis by combustion reactions and suggesting a statistical optimization for the process, this work applied the catalysts α-MoO_3_ and α/h-MoO_3_ obtained on a pilot scale through a combustion reaction in the production of biodiesel from waste cooking oil and ethanol. Using waste oil as a feedstock for biodiesel not only makes the process economically viable, but also avoids competition with edible oils, creating an environmentally friendly solution for disposing of waste cooking oil. The optimized parameters, including reaction time, type of catalyst, and alcohol/oil ratio, were determined using a 2^3^ experimental design, with 8 experiments and one replication of each, totaling 16 experiments.

## 2. Results

**Figure 1:**
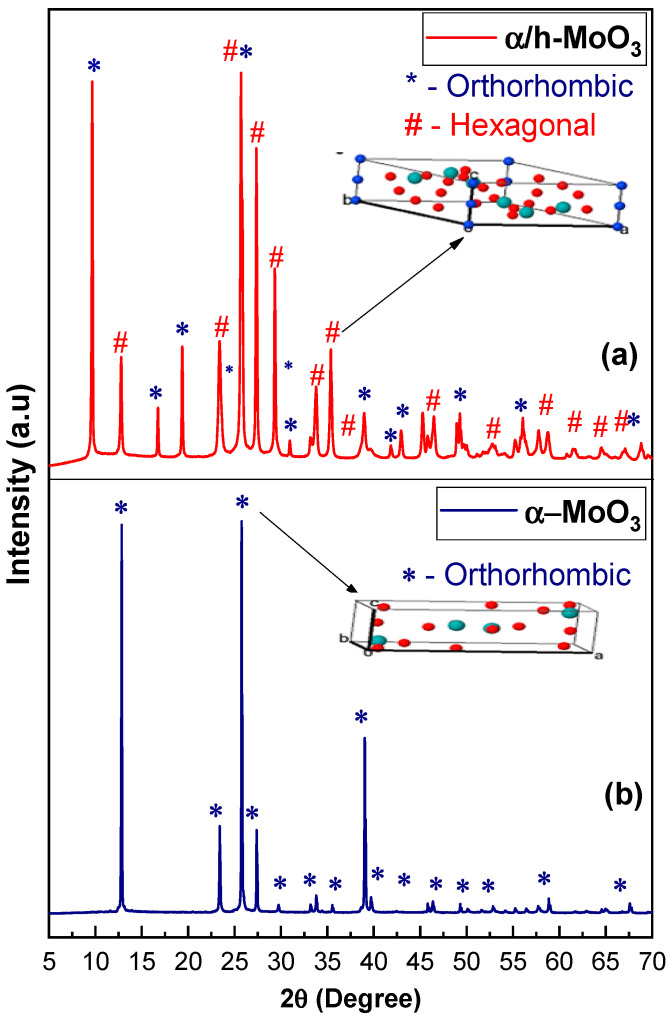
X-ray diffraction patterns for MoO_3_ catalysts: α/h-MoO_3_ (**a**) and α-MoO_3_ (**b**). Oxygen red; Molybdenum blue.

Figure 1a,b illustrates the X-ray patterns of α/h-MoO_3_ and α-MoO_3_ catalysts.

According to Figure 1, it is evident that the α-MoO_3_ catalyst exhibits a single-phase orthorhombic structure of MoO_3_, as indicated by the standard chart (PDF2(2019) 00-005-0508), and demonstrates a crystallite size of 84 nm with a crystallinity of 90%. These values surpass those observed for the α/h-MoO_3_ sample, which displays a biphasic structure comprising the hexagonal phase based on the standard reference (PDF2(2019) 00-065-0141), with the second phase corresponding to the orthorhombic crystalline structure of MoO_3_. The α/h-MoO_3_ sample exhibits a crystallite size and crystallinity of 33 nm and 88%, respectively. These findings suggest that the synthesis via combustion reaction to obtain both single-phase and two-phase MoO_3_ systems was efficient and achievable through a simple and cost-effective method, in contrast to similar studies in the literature utilizing the hydrothermal method for MoO_3_ synthesis. Additionally, successive calcinations post-synthesis were not necessary to achieve an orthorhombic single-phase product [34].

As for the actual density, for the α-MoO_3_ catalyst evidenced by the He pycnometry test, it was 4.5 g/cm^3^, showing little deviation in relation to the theoretical density revealed by the standard crystal chart PDF2(2019) 00-005-0508, which is 4.7 g/cm^3^, thus illustrating the excellent proximity of the material produced in this work to theoretical values. In contrast, the α/h-MoO_3_ catalyst sample presented a natural density of 3.8 g/cm^3^, a different value from that given in the PDF2(2019) 00-065-0141 standard letter of 7.9 g/cm^3^, a fact justified by the mixture of structures that make up the catalyst. Such behavior can be justified by the crystalline arrangement and intercalation of ions in the structure given to MoO_3_ materials, where a mixture of orthorhombic and hexagonal phases can cause a difference in the value of the sample’s absolute density. Concepts such as density are essential, as density measurements define the mass of catalytic solids that will be used in an industrial reactor [35].

**Figure 2:**
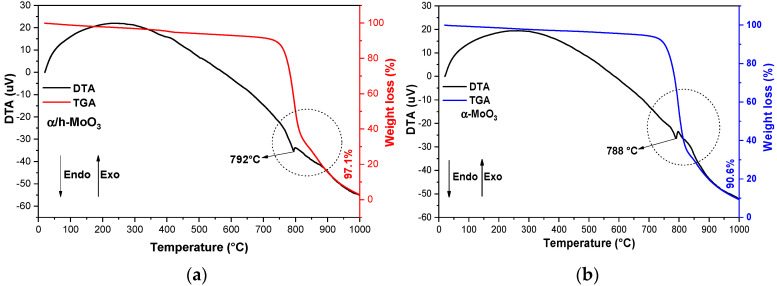
TGA/DTA curves for the catalysts: α/h-MoO_3_ (**a**) and α-MoO_3_ (**b**).

Figure 2 illustrates the thermal events observed from the TGA/DTA curves for the α/h-MoO_3_ and α-MoO_3_ catalysts. Based on this analysis, the temperatures (°C) of decomposition, phase transformation, and mass losses were determined.

The catalysts (Figure 2a,b) presented a similar profile to the curves for the TGA/DTA analyses, with thermal events of mass loss and an endothermic peak referring to phase transformation. This also illustrates the thermal stability of the catalysts obtained by combustion reaction at the imposed temperatures. Based on Figure 2a, thermal analysis (TGA) revealed thermal stability verified at temperatures up to ~697 °C, with three consecutive events of mass loss indicating the degradation process for the two-phase catalyst α/h-MoO_3_, in the temperature range between 372 and 896 °C, with a total mass loss of 97.1%. The DTA curve showed a discrete endothermic peak at temperatures around ~792 °C, which is possibly related to the melting point of the crystalline phase of the orthorhombic phase (α-MoO_3_), a phase present in the catalyst according to ray diffraction analysis. 

As shown in Figure 2b, thermal analysis (TGA) reveals that the thermal stability of the α-MoO_3_ catalyst is observed up to a temperature of ~725 °C; from then on, the degradation process begins through two events of sequential mass loss in the temperature range between 725 and 881 °C, with a total mass loss of 90.6%. The DTA curve illustrates a discrete endothermic peak around 788 °C, which is related to the melting point of the orthorhombic crystalline phase (α-MoO_3_). The results discussed in this work are very close and compatible with the literature [34,36,37] when studying thermal stability and catalytic characteristics of the α-MoO_3_ crystalline phase.

Table 1 describes the experimental values of the semiquantitative analysis of the oxides in the α/h-MoO_3_ and α-MoO_3_ catalysts, determined by EDX.

Based on Table 1, the percentages of oxides present given by X-ray fluorescence (EDX) for the α/h-MoO_3_ and α-MoO_3_ catalysts were (MoO_3_) 99.6% and (Fe_2_O_3_) 0.4%, similar values for the two catalysts under study. The EDX results confirm that molybdenum oxide is the majority element present in the catalysts obtained by combustion reaction, which corresponded to around ~99.6% of the total, followed by traces of iron oxide, which represented around 0.4%, possibly resulting from the preparation process by combustion reaction. The chemical analysis values expressed in this work agree with the studies of [38], when they studied MoO_3_ photocatalysts obtained by a method based on Pechini and applied in effluent treatment.

**Figure 3:**
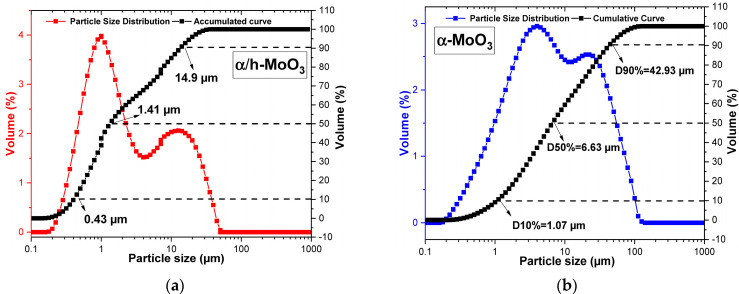
Particle size distribution for the catalysts: α/h-MoO_3_ (**a**) and α-MoO_3_ (**b**).

Figure 3 graphically illustrates the particle size analyses, which express the distribution values of the hydrodynamic diameters of the equivalent particles as a function of the cumulative volume for the α/h-MoO_3_ and α-MoO_3_ catalysts.

Analyzing Figure 3a,b, it can be seen that the α/h-MoO_3_ and α-MoO_3_ catalysts present a polymodal particle size distribution curve with a wide distribution range for sizes. In the α-MoO_3_ catalyst, it is possible to observe a concentration of particles between ~0.2 and 100 μm, with an average particle diameter of 15.43 μm being obtained. The accumulated values (black curve in the graph) illustrate an accumulation of particle sizes of 1.07 μm up to 10%, 6.63 μm up to 50%, and 42.92 μm up to 90%.

For the α/h-MoO_3_ catalyst, a concentration of hydrodynamic particle sizes slightly below 100 μm was observed, and a difference was observed between the synthesized samples. Another aspect that differs from the single-phase sample is the average particle size values of 7.7 μm and accumulated values of 0.43, 1.41, and 14.9 μm for the accumulated values D10, D50, and D90, respectively. The values observed for the α-MoO_3_ sample are lower or lower when compared to the two-phase catalyst. According to XRD analysis, this phenomenon can possibly be justified by the presence of two distinct morphologies of MoO_3_.

**Figure 4:**
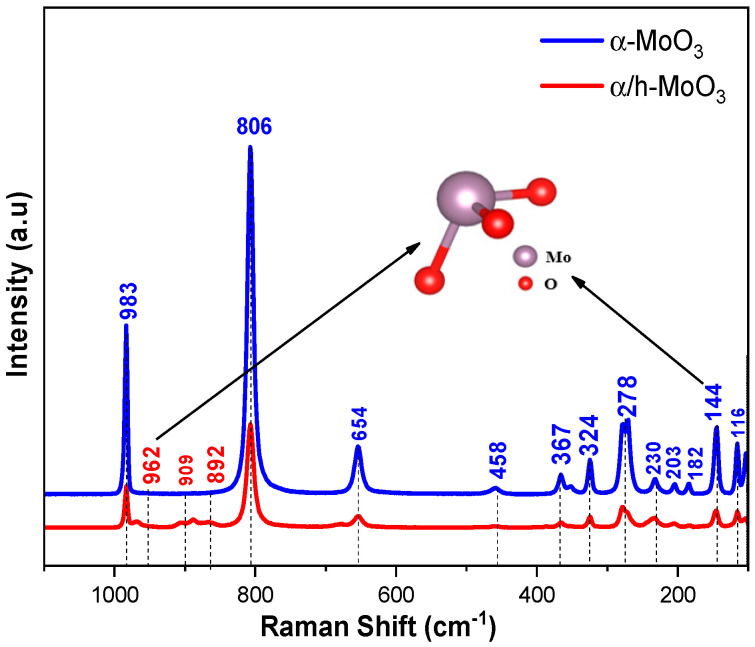
Raman spectroscopy of α-MoO_3_ and α/h-MoO_3_ catalysts.

Figure 4 illustrates the Raman spectra of MoO_3_ catalysts synthesized in this work.

The attributions observed in the Raman spectra (Figure 4) were based on those described in the literature [39,40,41,42]. The vibrational modes appearing around 600–1000 cm^−1^ correspond to the stretching vibrations of the MoO_6_ octahedron, while modes between 200 and 400 cm^−1^ originate from the bending vibrations of the MoO_6_ octahedron. Modes below 200 cm^−1^ are attributed to the deformation of the network of MoO6 octahedra, referring to the orthorhombic crystal configuration of MoO_3_ [39,42]. The bands observed in the range of 968–884 cm^−1^, seen as a distinguishing feature in the α/h-MoO_3_ sample, likely correspond to the elastic vibrations of the MoO_6_ octahedron in the hexagonal crystal structure of MoO_3_, corroborating the studies by Lopez et al. [42]. Such behavior was also noted by Zhang et al. [41], confirming the XRD results (Figure 1), which indicated that the catalyst is formed by two crystalline phases of MoO_3_.

The particle size values observed in this work differ from the recent literature consulted, which reports a nanometer scale for the particle size obtained by synthetic methods similar to that presented in this work [43,44,45].

**Figure 5:**
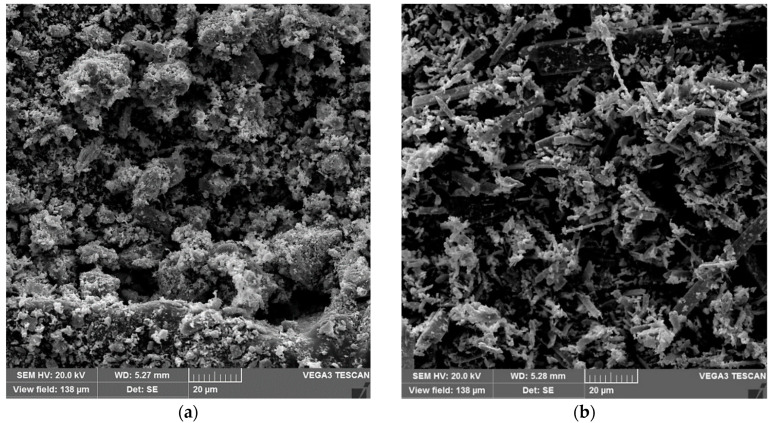
Morphologies obtained by SEM at 1500× for the support material α/h-MoO_3_ (**a**) and α-MoO_3_ (**b**)_._

Figure 5a,b illustrates the images obtained by scanning electron microscopy (SEM) of the α/h-MoO_3_ and α-MoO_3_ catalysts obtained by the combustion reaction method.

It is possible to observe that the α/h-MoO_3_ two-phase catalyst (Figure 5a) illustrates a mixed morphology with highly agglomerated short plates with apparent porosity possibly arising from the combustion synthesis process; the α-MoO_3_ single-phase catalyst (Figure 5b), illustrates elongated prismatic morphologies, with flat faces and well-defined edges, both with a wide range of cluster sizes, according to the granulometric distribution discussed and the recent literature studies [34].

**Figure 6:**
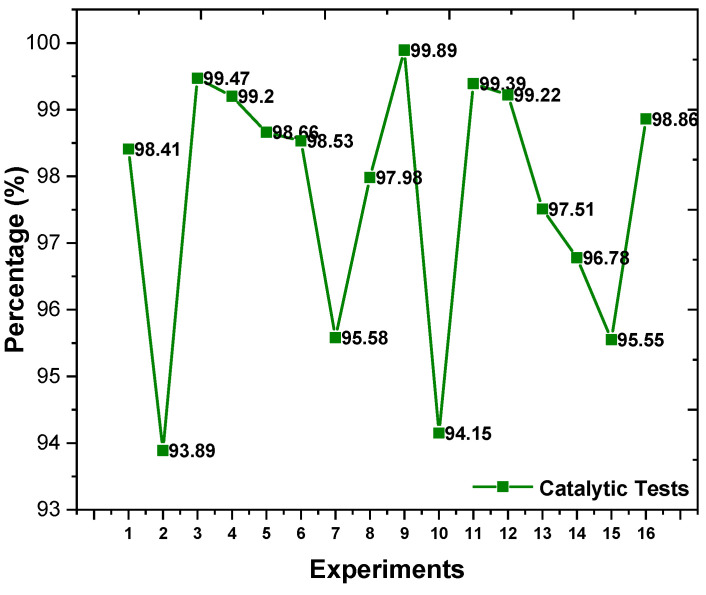
Percentage results of converting residual oil into ethyl esters obtained in the presence of α-MoO_3_ and h/α-MoO_3_ catalysts.

Table 2 describes and Figure 6 illustrates the experimental design response 2^3^ used to analyze and optimize the statistical data of biodiesel production.

It can be seen in Table 2 and Figure 6 that the α-MoO_3_ catalyst and an oil/alcohol ratio of 1/15 at an occurrence time of 60 min (Experiment 3) tend to provide higher conversion rates to ester. However, the catalysts synthesized by combustion reaction were active under all conditions tested and the conversions into fatty acid esters varied between 93 and 99%.

**Figure 7:**
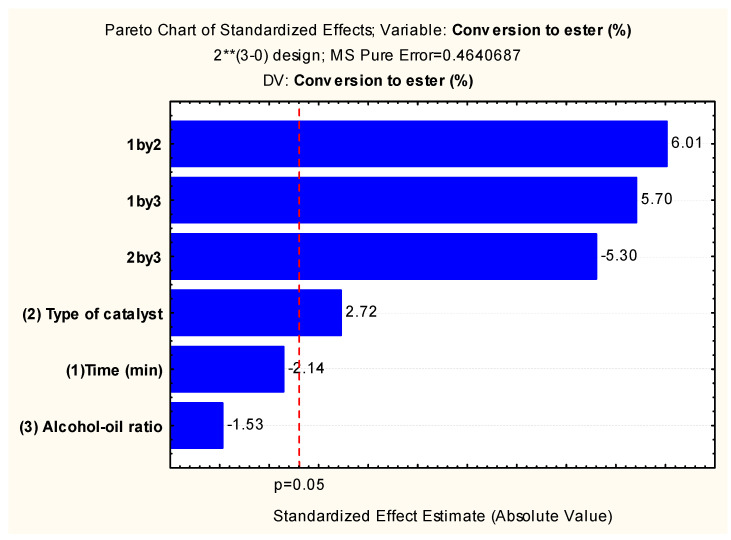
Pareto chart resulting from the 2^3^ factorial planning for converting waste oil into biodiesel. ** High.

The statistical study was carried out based on the observed reaction data (Table 2). The Pareto chart (Figure 7) was initially used as a response to the statistical analysis of experimental design 2^3^ to optimize the TES conditions of the residual oil catalyzed by α/h-MoO_3_ and α-MoO_3_.

It is possible to observe in Figure 6 that among the input variables, the most significant first order was the type of catalyst followed by all interactions of all other variables (oil/alcohol ratio and reaction time), with 95% reliability (*p* < 0.05) and with positive interference (+1) from the catalyst type variable, that is, the α-MoO_3_ catalyst presents a statistically significant interference to the process. The variables time and alcohol/oil ratio did not show a statistically significant effect. These observations corroborate the data in Table 2 and Figure 7, suggesting that the use of the single-phase catalyst significantly increases the conversion of residual oil into biodiesel.

**Figure 8:**
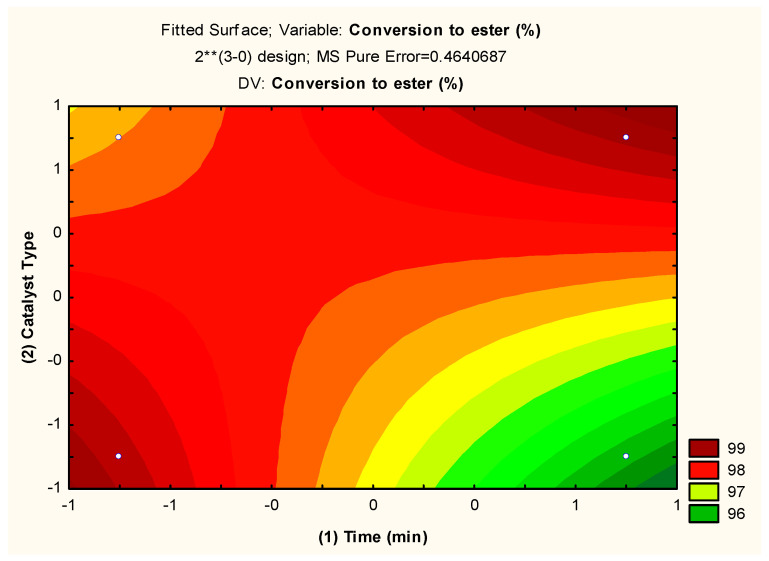
Conversion level curve of residual oil into biodiesel with the interaction between (2) type of catalyst and (1) time (min). ** High.

Level curves of the experimental planning response were also obtained, using as independent variables the type of catalyst and alcohol/oil ratio, and are illustrated in Figure 8.

The type of catalyst in the conversion reaction of residual oil into ethyl esters was better evaluated when using longer reaction times (+1) (120 min) and use of the single-phase catalyst (+1); at this point, the biodiesel conversion was at the maximum (close to 100%), corroborating what was observed in the Pareto chart (Figure 7).

In the region investigated, the response surface is satisfactorily described by the linear mathematical model given by Equation (1), which presented an R^2^ of 92% and which defines the plane represented in perspective on the level curve (Figure 8), based on the experimental planning carried out and which best describes the data collected and adjusted to the data in Table 2.

Z_(x,y)_ = 97.691875 − 0.365625 × x + 0.464375 × y + 1.024375 × x × y + 0.971875*0.*x − 0.903125*0.*y + 0 (1)

The data illustrated in Figure 7 and Figure 8 and the linear equation (Equation (1)) indicate that the statistical model adopted represents a good description of the experimental data related to the ethyl ester content in the reaction time of 1 h, oil/alcohol ratio 1/15, 4% catalyst, and temperature of 200 °C. These conditions were revealed as optimal by the statistical model used. The fit of the quadratic model was also tested by analysis of variance (ANOVA) according to Table 3.

The results expressed in the ANOVA (Table 3) presented a value for F_cal_/F_tab_ of 5.25 for the regression, indicating that the model was statistically significant, and the value for F_cal_/F_tab_ of lack of adjustment of 0.27, indicating that the model was predictive, so the F test revealed that the planning used was predictive and significant. Therefore, the regression model given by Equation (1) was a reasonable predictor of the experimental results, and the influenced factors were real with a confidence level of 95%, as already observed in Figure 6.

The optimal condition revealed by experimental planning 2^3^, which is in line with Figure 6, Figure 7 and Figure 8, Equation (1), and Table 2 and Table 3, is level -1 for the time variable (60 min), 1 for the type of catalyst (α-MoO_3_), and level −1 for the alcohol/oil molar ratio (1:15), indicating biodiesel conversions of ~99% with an overall desirability (~1) of 0.95, which illustrates that the optimization method used was efficient. Similar statistical behaviors were observed in the works of G. Novaes, T. Yamaki, F. de Paula, B. do Nascimento Júnior, A. Barreto, S. Valasques, and A. Bezerra [31], and Paula and Fernandes [46] when they used statistical planning to optimize variables that influence the performance of analytical methods and optimize water treatment, respectively.

**Figure 9:**
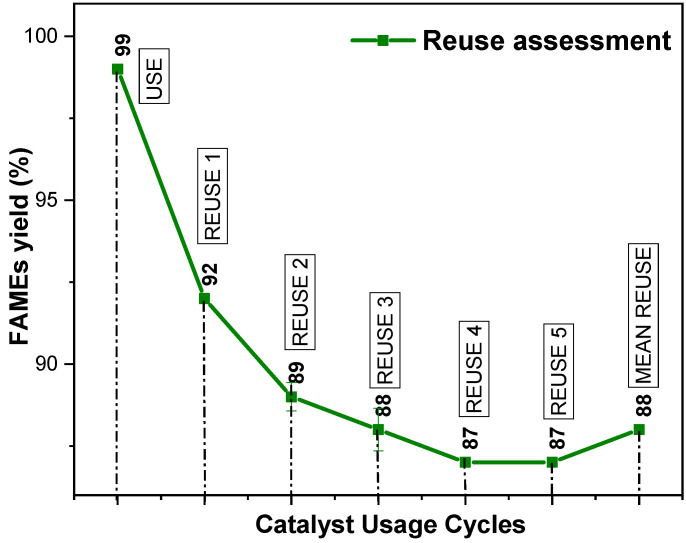
Results of reuse tests obtained for the α-MoO_3_ catalyst’ in TES reactions.

The optimal conversion condition for esters, the α-MoO_3_ catalyst, was recovered and reused in the TES reaction (conditions: 30% residual oil mass, 1-hour time, alcohol/oil ratio 15:1, 4% catalyst mass, and 200 °C). The conversion results obtained with the reuse are illustrated in Figure 9.

Based on Figure 9, the catalyst initially exhibited very high efficiency with a conversion to ethyl esters of 99% and a relatively low deviation of 0.04. This indicates that the catalyst was actively functioning in the first reaction it was used in.

As the catalyst was reused, a gradual decrease in its effectiveness was observed. Conversion decreased from 92 in the first reuse to 87 in the fourth and fifth reuses. Additionally, the deviations increased significantly over the reuses, indicating greater variability in the results as well as a change in its coloration over the reuse cycles.

Despite the decrease in effectiveness over the reuses, the average of the results still remains relatively high, around 88. However, the standard deviation remains at 0.14, suggesting that there is still a significant amount of variation in the results.

These data indicate that although the catalyst maintains a reasonably average effectiveness over the reuses, there is a slight trend towards decreased performance. This could be attributed to various factors such as accumulation of impurities, structural changes in the catalyst, or poisoning of the catalyst by undesired reaction products.

## 3. Materials and Methodology

### 3.1. Materials

The following materials were used in the synthesis of catalysts based on MoO_3_: Ammonium heptamolybdate—(NH_4_)_6_Mo_7_O_24_·4 H_2_O (99% purity, Sigma-Aldrich—Saint Louis, MO, USA), urea—CO(NH_2_)_2_ (99% purity, Dynamic —São Paulo, Brazil), nitric acid—HNO_3_ (65% purity, Nuclear—Rio de Janeiro, Brazil), ethylic alcohol—CH_3_CH_2_OH (99.5% purity, Dynamic), and *n*-hexane—C_6_H_14_ (99%, Neon São Paulo, Brazil). They were used as received without further purification. The residual oil used was collected in pastry shops in Campina Grande, located in Paraíba state, Brazil. Before experiments, the as-received residual oil was filtered (filter paper ₵15.00 ± 0.15 cm) to remove the suspended particulate matter. The oil showed fatty acid composition typical of soybean oil (12% of palmitic acid (16:0), 4% of stearic acid (18:0), 1% of oleic acid (18:1), 54% of linoleic acid (18:2), and 12% of linolenic acid (18:3)). The residual oil acidity was 6.79 0.05 mgKoH/g sample.

### 3.2. Methods

#### 3.2.1. Synthesis of Catalysts

The stoichiometry of the precursor reaction solution for MoO_3_ was calculated based on the total valence of oxidizing and reducing agents [47]. Ammonium heptamolybdate (AHM) ((NH_4_)_6_Mo_7_O_24_ 4H_2_O) was used as the metallic precursor, with the valences of the reactive elements as follows: C = +4; H = +1; N = 0; O = −2; and Mo = +6. Mo and O were considered oxidizing elements, while urea (CO(NH_2_)_2_) was considered the reducing agent (fuel). For maximum energy release, all oxygen content in the metallic precursor must oxidize, leading to a zero oxygen balance. Assuming a 1:1 stoichiometry, the synthesis requires an equivalent amount of “n” moles of fuel, where n = 0.03/6 = 0.005 moles of urea. Thus, the balanced theoretical chemical reaction for the synthesis of the MoO_3_ system can be expressed by Equation (2).

2[NH_4_]_6_Mo_7_O_24_(aq) + 4H_2_O(aq) + 6CO(NH_2_)_2_(aq) ↔ 14MoO_3_(s) + 18NH_4_(g) + 4H_2_O(g) + 6CO_2_(g) +3N_2_(g) (2)

The catalysts were synthesized via combustion reaction in a stainless steel container, coded P08, and prepared to obtain powders with a batch production of 20 g, following the device and methodology patent INPI: BR10 2021 018179-6 [48] and the combustion reaction synthesis architecture described in the patent device INPI: BR 10 2012 002181-3 [49].

#### 3.2.2. Catalytic Test

The performance of MoO_3_ catalysts was evaluated in the synthesis of biodiesel using waste oil via TES reaction. The catalytic tests were conducted in a stainless-steel pressurized system reactor (Parr 4848) with a capacity of 100 mL, mechanical stirrer, time and temperature controller, and pressure indicator. After the TES reactions, the catalysts were separated from the products in a centrifuge (3500 rpm) and subsequently purified with n-hexane, washed with distilled water at ~70 °C, and then dried in an oven at 110 °C/24 h. The biodiesel produced was washed with distilled water at ~70 °C and dried in an oven at 110 °C for 30 min with manual stirring at 5 min intervals.

#### 3.2.3. Catalyst Reuse

After the reaction, the catalysts were collected, washed with n-hexane, and dried in an oven at 110 °C for 24 h and then reserved for sequential reuse. The reuse tests were accomplished under the best reactive conditions established from the results of the catalytic tests.

#### 3.2.4. Statistical Analysis

For the analysis and optimization of the biodiesel synthesis process from waste oil, a 2^3^ factorial experimental design was prepared with 8 experiments with one replication (duplicate injection for gas chromatography analysis), totaling 16 random experiments, which were analyzed using a Pareto chart, level curves, and ANOVA table, and were evaluated in the *Statistic 7.0* program. Table 4 describes the input levels and variables for the proposed experimental design.

Time, type of catalyst, and oil/alcohol ratio are considered in the factorial experimental design 2^3^. The two levels for the selected factors were determined from preliminary exploratory experiments and assistance from the recent published literature [34,50,51]. The planning matrix (Table 5) was obtained from the choice of variables and levels with the help of the *Statistic 7.0* software, which was used to analyze the experiments using level curves, the ANOVA table, and the Pareto.

The conversion of residual oil into biodiesel (Y) was used as a response to determine the optimized parameters. The effect of independent factors on dependent factors was analyzed using a linear equation (Equation (3)), following the suggestion of a linear configuration for the proposed planning:

(3) $$Y=\alpha 0+\Sigma_{i=1}^{k}{\alpha \mathrm{iXi}\;\Sigma }_{i<1}^{k}\alpha \mathrm{ijXiXj}+e$$

where Y is the ester content response variable; α0 is the compensated term; αi is the first order linear coefficient; αij is the linear interaction coefficient between variables, and refers to the pure error associated with the experiments; and Xi and Xj are input variables.

#### 3.2.5. Characterizations

The α-MoO_3_ catalyst was characterized by X-ray diffraction (XRD) using a BRUKER (Billerica, MA, USA) X-ray diffractometer (model D2 PHASER, Cu-Kα radiation), operating with 30 kV and 10 mA, with copper Kα radiation source (*k*α = 1.54056 Å). The sweep range used at 2θ = 10° at 70°, with an angular step of 0.016° and a counting time of 1.000 s per step. The crystalline phases identification were carried out from the ICDD crystallographic records of the PDF2/2019 database with the aid of *DiffracPlus* Suite Eva software V 7, which was also used to obtain the crystallinity values and the size of the crystallite (calculated with the aid of the Scherrer equation) [52].

Raman spectra were recorded on a RENISHAW (West Dundee, IL, USA) spectrophotometer (model InVia Raman microscope) using an Ar^+^ laser, with a power of 100 mW and a wavelength of 514 nm. The 50× objective was used. Raman spectra were obtained under room temperature, in the frequency ranges of 90–1200 cm^−1^.

The actual density value of the catalyst was obtained through the analysis with a Quantachrome Corporation (Boynton Beach, FL, USA) Upyc 1200e v5.04 Pycnometer operating with helium gas (He). Thermogravimetric analysis (TGA/DTA) was evaluated using a Shimadzu (Tokyo, Japan) TA 60H simultaneous thermal analysis system, with a heating rate of 12.5 °C/min under air atmosphere 100 mL/min (maximum temperature 1000 °C). The semi-quantitative analysis of the oxides and elements present in the samples were determined by energy dispersive X-ray fluorescence spectroscopy, model EDX-720, from Shimadzu. The laser diffraction particle size analysis uses the liquid phase particle dispersion method associated with an optical measurement process through laser diffraction on the Mastersizer 2000 equipment from Malvern (Worcestershire, UK). The morphological aspects of the catalyst sample were acquired by scanning electron microscopy (SEM), brand Tescan (Brno, Czech Republic), model Vega3.

The percentages of ethyl esters were determined by gas chromatography using a VARIAN (Las Vegas, NV, USA) 450c instrument chromatograph with flame ionization detector and capillary column as stationary phase (Varian Ultimetal “Select Biodiesel Glycerides RG”; dimensions: 15 m × 0.32 mm × 0.45 mm). The initial injection and oven temperatures were 100 °C and 180 °C, respectively, and the detector operated at 380 °C.

## 4. Conclusions

Combustion synthesis has proven to be highly effective in producing catalysts. MoO_3_-based catalysts were obtained in orthorhombic and hexagonal phases, with crystal sizes ranging from 35 to 81 nm and crystallinity between 88% and 90%. Additionally, they exhibited remarkable thermal stability, enduring temperatures up to 800 °C. Molybdenum oxide emerged as the predominant component in these catalysts.

During catalytic activity tests, the catalysts showed efficiency under all evaluated reaction conditions. Remarkably, the monophasic α-MoO_3_ catalyst displayed slight superiority in catalytic activity, achieving a 99% conversion for ethyl esters at 200 °C for one hour, with a catalyst concentration of 4% by mass of ethanol/oil, and a molar ratio of 15:1.

Furthermore, this monophasic catalyst proved to be recyclable, maintaining a lifespan of up to six cycles with an average reuse of 88%. The use of factorial design allowed for a more in-depth analysis of the involved variables (catalyst type, alcohol/oil ratio, and time), revealing that catalyst type had the most significant influence on the residual oil’s TES. The adopted statistical model was both significant and predictive, with a significance level of 95%.

The optimal conditions determined by experimental design resulted in high conversions to biodiesel, demonstrating the efficacy of the linear optimization employed. These findings align with the existing literature and underscore the viability of using α-MoO_3_ catalysts in biodiesel production from residual oil, employing optimization strategies to achieve the best catalytic outcomes possible. The catalysts developed in this study exhibit a high potential for industrial applications, especially when compared to residual oil and the ethanol routes, thus potentially making positive contributions to the environment and global society at large.

## Figures and Tables

**Table 1:** Percentages of oxides present by X-ray fluorescence (EDX) for the α/h-MoO_3_ and α-MoO_3_ catalysts.

Oxides Present	Catalysts	
	α/h-MoO_3_	α-MoO_3_
MoO_3_	99.79%	99.57%
Fe_2_O_3_	0.21%	0.43%

**Table 2:** 2^3^ factorial experimental design response obtained for the random variables time, type of catalyst, alcohol/oil ratio, and conversion to ester.

Experiment	(1) Time (min)	(2) Type of Catalyst	(3) Alcohol/Oil Ratio	Y = Conversion to Ester (%)
1	60	α/h-MoO_3_	1/15-	98.41
2	120	α/h-MoO_3_	1/15	93.89
3	60	α-MoO_3_	1/15	99.47
4	120	α-MoO_3_	1/15	99.20
5	60	α/h-MoO_3_	1/20	98.66
6	120	α/h-MoO_3_	1/20	98.53
7	60	α-MoO_3_	1/20	95.58
8	120	α-MoO_3_	1/20	97.98
9(R) *	60	α/h-MoO_3_	1/15	99.89
10(R) *	120	α/h-MoO_3_	1/15	94.15
11(R) *	60	α-MoO_3_	1/15	99.39
12(R) *	120	α-MoO_3_	1/15	99.22
13(R) *	60	α/h-MoO_3_	1/20	97.51
14(R) *	120	α/h-MoO_3_	1/20	96.78
15(R) *	60	α-MoO_3_	1/20	95.55
16(R) *	120	α-MoO_3_	1/20	98.86

* (R) Replicate. Highlighted the best conversions in planning ester.

**Table 3:** ANOVA to optimize biodiesel production from waste oil using an α-MoO_3_ catalyst.

ANOVA Table				
Source of Variation	Quadratic Sum	Degrees of Freedom	Quadratic Mean	
Regression	51.63	6	8.60	F_cal._ of regression 17.71
Waste	4.37	9	0.49	F_calc._ of (Lack of fit) 1.42
Lack of adjustment	0.66	1	0.66	
Pure error	3.71	8	0.46	
Total	56.00	15		
F_tabelado_ REG	3.37	F_cal_/F_tab_ (Regression)	5.25	
F_tabelado_ da F. Aj	5.32	F_cal_/F_tab_ (Lack of fit)	0.27	
%Mx. explained	92.19	Model Statistics		
%Mx. explainable	93.37	R^2^ (%)	92.19	
R^2^	0.92	R^2^ adjusted (%)	76.44	
Fit quality	0.76	F_calc_/F_tab_ (regression)	5.25	
S (standard error of regression)	0.70	F_calc_/F_tab_ (lack of fit)	0.27	
		Regression standard error	0.70	

**Table 4:** Variables and input levels proposed for experimental planning 2^3^.

	Levels	
**Variables**	−1	+1
(1) Time (min)	60	120
(2) Type of catalyst	α/h-MoO_3_	α-MoO_3_
(3) Alcohol/oil ratio	15:1	20:1

**Fixed conditions:** Temperature 200 °C, 30 g of residual oil, and 4% catalyst.

**Table 5:** 2^3^ factorial planning matrix with replication for the catalytic test experiments.

Experiment	(1) Time (min)	(2) Type of Catalyst	(3) Alcohol/Oil Ratio
1	−	−	−
2	+	−	−
3	−	+	−
4	+	+	−
5	−	−	+
6	+	−	+
7	−	+	+
8	+	+	+
9(R) *	−	−	−
10(R) *	+	−	−
11(R) *	−	+	−
12(R) *	+	+	−
13(R) *	−	−	+
14(R) *	+	−	+
15(R) *	−	+	+
16(R) *	+	+	+

* (R) Replica of injection in the chromatograph.

## Data Availability

Data are contained within the article.
